# VDJSeq-Solver: In Silico V(D)J Recombination Detection Tool

**DOI:** 10.1371/journal.pone.0118192

**Published:** 2015-03-23

**Authors:** Giulia Paciello, Andrea Acquaviva, Chiara Pighi, Alberto Ferrarini, Enrico Macii, Alberto Zamo’, Elisa Ficarra

**Affiliations:** 1 Department of Control and Computer Engineering, Politecnico di Torino, Torino, Italy; 2 Department of Pathology and Diagnostics, University of Verona, Verona, Italy; 3 Department of Pathology, Children Hospital Boston, Harvard Medical School, Boston, USA; 4 Department of Biotechnology, University of Verona, Verona, Italy; University of Science and Technology of China, CHINA

## Abstract

In this paper we present VDJSeq-Solver, a methodology and tool to identify clonal lymphocyte populations from paired-end RNA Sequencing reads derived from the sequencing of mRNA neoplastic cells. The tool detects the main clone that characterises the tissue of interest by recognizing the most abundant V(D)J rearrangement among the existing ones in the sample under study. The exact sequence of the clone identified is capable of accounting for the modifications introduced by the enzymatic processes. The proposed tool overcomes limitations of currently available lymphocyte rearrangements recognition methods, working on a single sequence at a time, that are not applicable to high-throughput sequencing data. In this work, VDJSeq-Solver has been applied to correctly detect the main clone and identify its sequence on five Mantle Cell Lymphoma samples; then the tool has been tested on twelve Diffuse Large B-Cell Lymphoma samples. In order to comply with the privacy, ethics and intellectual property policies of the University Hospital and the University of Verona, data is available upon request to supporto.utenti@ateneo.univr.it after signing a mandatory Materials Transfer Agreement. VDJSeq-Solver JAVA/Perl/Bash software implementation is free and available at http://eda.polito.it/VDJSeq-Solver/.

## Introduction

The B-cells and T-cells of jawed vertebrates possess unique genomes due to structural rearrangements of B-cell receptor (BCR) and T-cell receptor (TCR) for antigens, caused by complex and dynamical rearrangement events involving several variable (V), diversity (D) and joining (J) gene segments [1, 2]. The antigen receptors on B-cells are multiprotein complexes made up of clonally variable antigen-binding chains called Immunoglobulin (IG) chains associated with the coreceptor CD79A and CD79B proteins. Every chain is characterised in its amine-terminus (N-terminal) portion by a variable amino acid sequence that is involved in specific antigen binding and in its carboxy-terminus (C-terminal) region by a constant part that defines the class and effector function of the antibody molecule. Variable regions of Immunoglobulin Heavy (IGH) and Immunoglobulin Light (IGL) chains of BCR are assembled respectively from germline V, D, J and V, J segments thanks to a site-specific reaction called V(D)J recombination that involves the developing of B lymphocytes [3, 4].

In particular, for what is concerning IGH, several different mechanisms generate the variable region diversity with respect to V(D)J recombination. The combinatorial diversity that comes from the different rearrangements of the V, D and J germlines is further improved by the diversification of the junction between the three segments during the V(D)J recombination. This process is indeed characterised by the introduction of nucleotides by the Terminal deoxynucleotidyl Transferase (TdT) [5] that follows the deletion of nucleotides at the 3’ end of the V gene segment, at the 5’ end of the J gene segment, and at both the ends of the D gene segment which recombine. In absence of this last process very short inverted sequences, called palindromic-regions, can be found at the V(D)J junction.

This diversity determines the huge variability of interactions possible between antigens and antigen receptors, that is one of the pillars of the adaptive immune response. B-cells and T-cells are therefore different from other cells in the fact that their genomes bear a genomic birthmark of diversity. They can expand under specific conditions (e.g. antigen encounter) and form monoclonal populations bearing identically rearranged gene segments [6, 7]. These clonal populations are usually under tight control mechanisms. However, under special occasions they might expand to an extent which causes a disease, such as in autoimmune disorders, leukemias and lymphomas [8].

Recently different studies have been devoted to the characterisation of the BCRs in different pathologies leading, for example, to the discovery of biases in the usage of specific IGH [9–16] or IGL gene segments [17, 18] with respect to the normal expected distribution and to the correlation of the clinical course of the disease with the rate of somatic hypermutation of BCR gene segments [19, 20]. PCR-based clonality tests are nowadays very popular in diagnostic hematopathology, being able to detect abnormal expansion of single clonal populations in a normal polyclonal lymphocyte population. Clonality tests are however characterised by remarkable drawbacks such as the difficulty in designing proper primer sets capable to amplify all possible gene segments rearrangements and the intrinsically non-quantitative nature of PCR techniques.

To overcome these limitations in this paper we present a methodology and the related tool, called VDJSeq-Solver, that exploits Next-Generation Sequencing (NGS) [21] and more specifically RNA Sequencing (RNA-Seq) [22], to identify and quantify the clonal RNA fragments present in a sample thanks to the detection of the relative V(D)J rearrangement, even if characterised by mutated nucleotides.

The target of the methodology is: i) To detect abnormal lymphocyte populations in a sample, even when they are present in very low amounts; ii) to find specific IGHV gene segments that may be correlated with clinical subsets bearing a different prognosis as found to be significant in B-cell Chronic Lymphocytic Leukemia (B-CLL) [9]; iii) to provide a system for disease monitoring, including minimal residual disease detection. Finally, the proposed methodology poses the way to the development of powerful diagnostic classification by coupling with disease-specific signatures. Also, this approach could be applied to the detection of the whole IG repertoire (and possibly TCR repertoire) to define the immunological picture of a sample, be it neoplastic or not.

Based on NGS technology, we identify clonal lymphocyte populations (also in the context of a polyclonal background) by quantifying the amount of RNA expressed by the gene segments rearranged in the neoplastic clone with respect to the total amount of RNA expressed by the other BCR gene segments. This quantification is possible by counting the reads (i.e. the elementary sequence elements of NGS technologies) mapping on a reference represented by the rearranged gene segments. At the same time the methodology provides precise information concerning the rearranged BCR gene segments of the dominant clone.

However, the process of mapping reads on rearranged gene segments poses a number of challenges, primarily because of the diversity of the references on which the alignment is made, determined by the rearrangements of gene segments taken by V, D, J regions and by the inserted or deleted nucleotides due to enzymatic processes.

Various tools [23–32] have been developed in the last years with the main purpose of finding the best match between a rearranged sequence and the V, D and J germlines: All of them try to assign a specific V, D and J alleles to a unique sequence, extracted in laboratory via Polymerase Chain Reaction (PCR) or via high-throughput sequencing experiments [33, 34].

IMGT/V-QUEST [23, 26–28] is probably the most known tool because it is the first automatic tool developed to analyse IG V(D)J regions. IMGT/V-QUEST identifies the V, D and J gene segments by alignment with the germline IG and TCR gene sequences of IMGT reference directory. It analyses batches of sequences (up to 50) in a single run. IMGT/V-QUEST describes the V region mutations and identifies the hot spot positions in the closest germline V gene. It is able to detect insertions and deletions in the submitted sequences by reference to the IMGT unique numbering. Furthermore it integrates IMGT/JunctionAnalysis for a detailed analysis of VJ and V(D)J junctions and IMGT/Automat for a full VJ and V(D)J region annotation.

IMGT/JunctionAnalysis [30, 31] tries to overcome the problems related to the identification of the D allele and the nucleotides deleted or introduced by the specific processes proper of the V(D)J recombination. The junction is here defined as the region starting at the second conserved cysteine of the V region at position 104 (2nd-CYS) and ending with the conserved tryptophan (J-TRP for the IGH chains) or the conserved phenylalanine (J-PHE for the IGL chains or the TCR chains) at position 118. IMGT/JunctionAnalysis searches the constitutive regions of the junction by comparing the user sequence with the IMGT reference directory, but V and J allele names have to be identified thanks to IMGT/V-QUEST.

JOINSOLVER [29] deals with the difficulty of D gene segment assignments giving a higher score for longer consecutive nucleotides matches, but searches for two relatively conserved motifs *TAT TAC TGT* and *C TGG GG* to find the extreme points of the Third Complementarity Determining Region (CDR3) that is the most variable part of BCR and TCR.

SoDA [32] is another tool developed for deciphering BCR and TCR gene segments composition. Initially the set of possible V, D and J gene segments is chosen thanks to independent unconditional pairwise alignments between the target gene and each candidate gene, in particular for what is concerning D segments each candidate is evaluated by alignment against the part of the target sequence between the V conserved CYS and the conserved J-TRP or J-PHE. In the second phase of the pipeline all the gene segments are at the same time aligned against the previous identified sets.

Programs such as VDJSolver [25] and iHMMune-align [24] apply instead statistical models to obtain the optimized parameters fitting to the rearranged sequence. These methods represent an alternative way to identify the rearrangement but the model robustness heavily depends on the quality and diversity of the training data sets to obtain satisfactory performances even if applied on different kind of antibodies.

Furthermore, in order to analyze a larger set of input sequences deriving from the high throughput and deep sequencing of IG and TCR, a web portal called IMGT/HighV-QUEST has been recently proposed [28, 33, 35, 36]. It is implemented for the analysis of long (about 400 nt) V(D)J recombined sequences and allows among its features the identification of the closest V, D and J genes and alleles, the IMGT/JunctionAnalysis application, the description of mutations and the characterisation of IMGT clonotypes. With respect to IMGT/HighV-QUEST, that works with longer sequences generally harbouring the entire V(D)J recombination, the proposed methodology is able to reconstruct the main clone rearranged sequence using a set of relatively short RNA-Seq paired-end reads. Moreover, no limitations concerning the maximum number of input reads is imposed by VDJSeq-Solver tool.

Summarising, with respect to currently available pipelines and tools, the method proposed in this work allows extracting clonality information from *primer-free* (i.e. not previously PCR-amplified using IG-specific primers) RNA-Seq data, specifically 100 base pair (bp) long paired-end reads. While RNA-Seq data must be available for the sample of interest in order to detect existing V(D)J rearrangements, it is not necessary to construct ad hoc primers for IG, usually accounting for different amplifications among sequences.

Starting from a set of paired-end RNA-Seq reads, derived from the sequencing of mRNA neoplastic cells, our pipeline aims at identifying the main clone that characterises the tissue of interest by detecting the most abundant V(D)J rearrangement. Secondly, considering the amount of reads that are mapped on the V, D and J gene segments involved in the identified rearrangement, the specific sequence of the clone is reconstructed. Being extracted from real data the obtained sequence is capable to account for the modifications introduced by the enzymatic processes.

VDJSeq-Solver pipeline has been applied on twelve Diffuse Large B-Cell Lymphoma (DLBCL) RNA-Seq samples from *The Cancer Genome Atlas (TCGA)* and five Mantle Cell Lymphoma (MCL) RNA-Seq samples in order to identify the main clone. Furthermore the main clone V(D)J rearranged sequence has been retrieved for the last five samples. These recombined sequences, obtained as said from the reads and so capable to account for the nucleotides introduced or deleted by the enzymatic processes, have been also evaluated thanks to freely available online tools as it will be explained in the *Results* Section. Our results show that this approach is feasible, and pose the methodological basis for the development of a diagnostic approach relying on this kind of output. VDJSeq-Solver is implemented in Java/Perl/Bash languages and runs on a standard Linux machine.

## Materials and Methods

The objective of VDJSeq-Solver pipeline is twofold. First, the identification of the main clone, as lymphoid neoplasms, a clonality is indeed proven by the amplification of a single rearrangement of their BCR gene segments. Second, retrieval of the specific recombination sequence for the identified recombination. In this phase we consider that the diversity of each sequence results from: i) The addition of nucleotides by the TdT at the junction of the V, D and J gene segments during the rearrangement; ii) the deletion of nucleotides at the 3’-end of the V gene segment, at the 5’-end of the J gene segment and at both the ends of the D gene segment which recombine; iii) the presence of very short regions called palindromic-regions at the V(D)J junctions.

**Fig 1:**
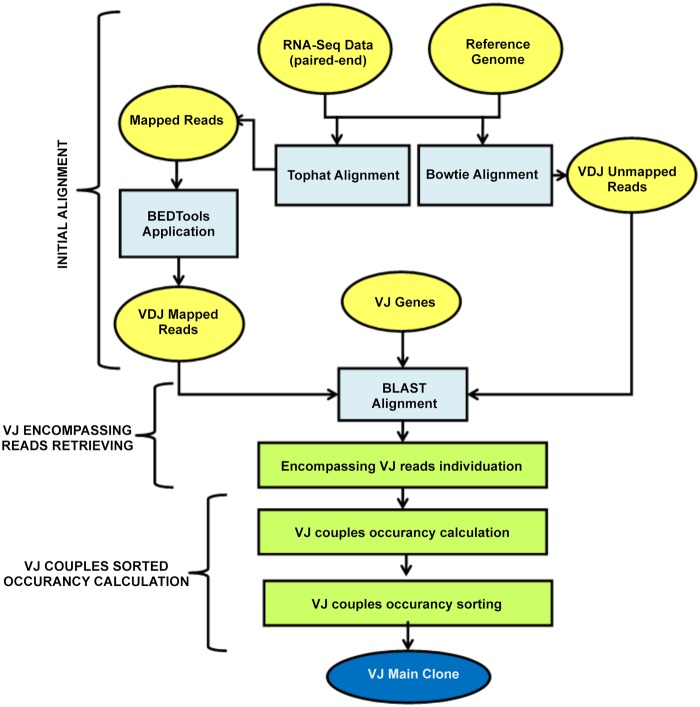
VJ alleles individuation. In Figure are detailed the different activities, as well as inputs and outputs, executed during the VJ alleles research. With yellow ellipses are depicted respectively input and output data, with exception of the final result that is highlighted with a dark blue rectangle. With light blue boxes are represented the operations performed taking advantage of freely available tools and finally with light green rectangles those performed thanks to ad hoc developed programs.

**Fig 2:**
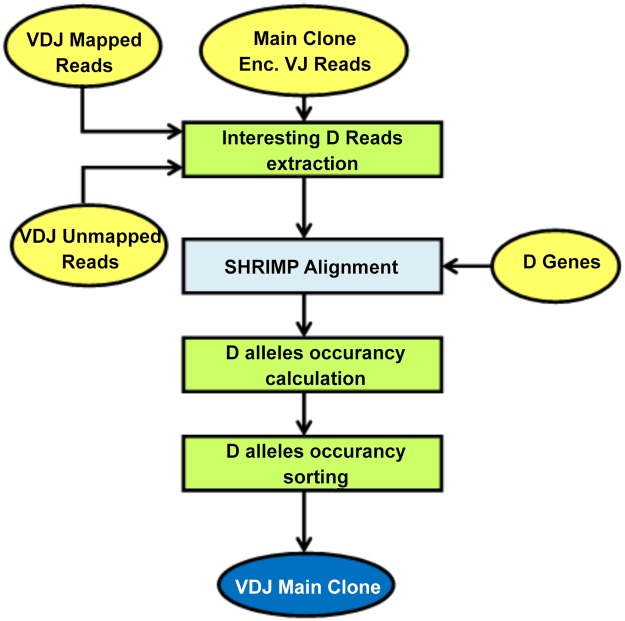
D alleles individuation. In Figure are detailed all the activities executed during the D alleles research, as well as inputs and outputs. With yellow ellipses are depicted respectively input and output data, with exception of the final result that is highlighted with a dark blue rectangle. With light blue boxes are represented the operations performed taking advantage of freely available tools and finally with light green rectangles those performed thanks to ad hoc developed programs.

**Fig 3:**
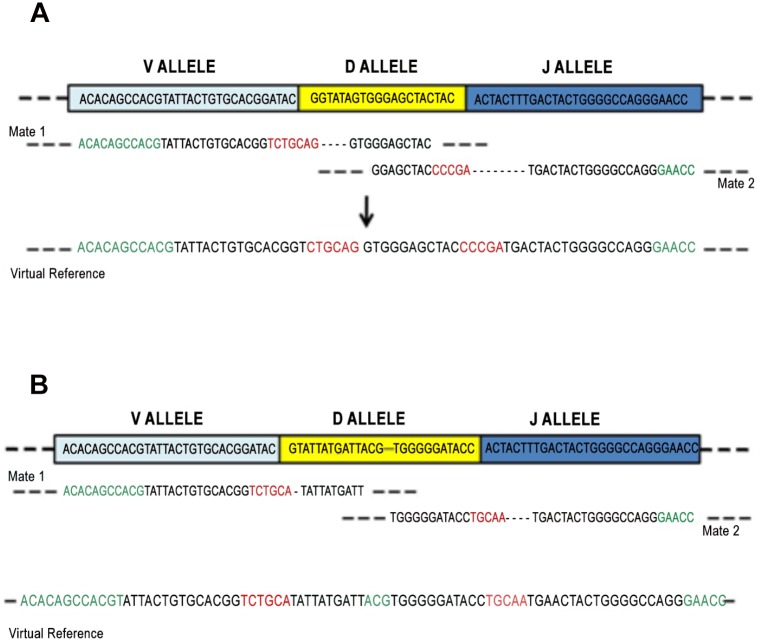
V(D)J sequences retrieving. In Figure are represented two situations that might occur during the V(D)J main clone sequence creation: i) The two mates are partially overlapped in the D region (A); ii) the two mates are not overlapped in the D region (B). In green letters are represented the extensions in both 5’ and 3’ directions of the V, D and J gene segments previously identified as rearranged, in black characters the portions of the considered mates that have been mapped on the V, D or J gene segments of interest and in red characters the nucleotides, extracted from the considered mates, that have been assigned to the VD and DJ junctions.

The proposed flow is mainly composed of two building blocks. *Main Clone Identification* (see Figs. 1 and 2) identifies the main clone contained in the tissue under study. *V(D)J Sequence Retrieving* (see Fig. 3A and B) extracts the sequence of the specific recombination.

### Main Clone Identification

The individuation of the main clone is conducted in two phases. The first, named *VJ alleles individuation* and shown in Fig. 1, aims to identify the V and J gene segments from which the variable regions of the different clones are arranged and to score each VJ rearrangement on the basis of the number of reads supporting it. The second, named *D alleles individuation* and represented in Fig. 2 recognizes, for the most supported VJ couples identified before, the D allele introduced during the recombination process.

#### VJ alleles individuation

The individuation of the VJ recombinations in the sample under study is conducted following the steps shown in Fig. 1 and detailed below.

##### Initial Alignment

The starting point for determining the list of the VJ rearrangements in the sample under study is the alignment of its short RNA-Seq paired-end reads to the reference genome (we used GRCh37 assembly). The alignment is performed taking advantage of two different tools in order to obtain two datasets. The first dataset contains those reads that are not mapped on the genome due splicing events among the V, D and J gene segments involved in the recombination (*VDJ unmapped* reads). The second dataset contains instead those reads that are mapped on the V, D and J gene segments, the *VDJ mapped* reads.

The first alignment is performed taking advantage of Bowtie tool [37], whereas the second uses TopHat aligner [38] that reports variable length alignments due to the presence of junction breakpoints caused by splicing events. Bowtie alignment is performed in -v mode on the two mates separately, imposing a maximum number of mismatches equal to 2. It is furthermore specified to provide up to 10 valid alignments in the best alignment “stratum” and to refrain from reporting any alignments for reads having more than 10 reportable alignments. The unmapped reads deriving from this alignment procedure constitute the first dataset of interest.

Concerning TopHat mapping parameters, a mate inner distance of 210 and a standard deviation for the distribution on inner distances between mate pairs of 30 were selected. The maximum number of mismatches allowed in the so called anchor region of a spliced alignment was fixed to 2. The selected parameter values were able to guarantee satisfactory performances both in terms of accuracy and computational costs of the alignment. The second dataset of interest is obtained by extracting the reads mapping in the V(D)J IGH locus from the whole set of mapped reads output of Tophat aligner. This operation is executed taking advantage of the BEDTools Utilities [39].

##### VJ encompassing reads retrieving

*VDJ mapped* and *VDJ unmapped* reads are aligned against V and J gene segments using Blast [40] blastn program in order to retrieve only those mates mapped on the 272 V or the 16 J genes proper of the IGH chain locus. Because of the smaller size of the dataset obtained at the end of the previous alignment phases, it is now computationally feasible to use a local alignment tool such as Blast to obtain a more accurate mapping. We identify a VJ recombination if, for a given read, a mate is mapped on a V gene segment and the other mate is mapped on a J gene segment. We call these reads *VJ encompassing* reads. Note that, due to the remarkable polymorphism occurring among the considered gene segments [41], the same read can define multiple VJ couples.

##### VJ couples sorted occurrence calculation

Each of the identified VJ couples is scored on the basis of the number of encompassing reads supporting the recombination. In this phase, if a given read supports the same VJ recombination at different positions on the two gene segments of interest, that read will be counted only once in the quantification of that specific recombination. This prevents the overestimation of supporting reads caused by multimapping due to homologies inside the same gene segments. On the other side, if the same read supports more than one recombination (due to polymorphisms and homologies [41]), this will be counted in the quantification of all the recombinations. This is a conservative approach, as it is not possible in this phase to make a decision about the correct assignment.

A sorting based on the number of *VJ encompassing* reads supporting the detected recombinations is then performed. At the end of this phase a list of all the identified clones is given. The most supported couple is defined as the one characterising the main clone.

#### D alleles individuation

For the most supported VJ couples, the recombining D gene segment is identified in this phase. As shown in Fig. 2, only the mates belonging to a *VJ encompassing* read that do not map totally on the V or J gene segments are considered. These mates are aligned using Shrimp [42] on the D gene segments by imposing a minimum perfect alignment length of 10 nucleotides (seed sequence) and only one reported alignment for each mate. Shrimp is suitable to this phase because it allows to map reads to a genome even in presence of a considerable amount of polymorphisms, which is the case for D gene segments. A sorting based on the occurrence of each D gene segment is performed and the most supported among them is selected to be associated to the considered VJ couple.

### V(D)J Sequence Retrieving

In order to reconstruct the recombined sequence for the V(D)J rearrangement of the main clone, a virtual reference that takes into account the nucleotides introduced or deleted by the enzymatic processes have to be created. Only those reads for which both the mates have been partially mapped on the D gene segment are considered to build the virtual reference.

These reads may fall in two cases: i)The two mates are partially or totally overlapped in the D region (see Fig. 3A); ii) the two mates are not overlapped in the D region (see Fig. 3B).

In both the cases the reference is built by extracting from the reads only those fragments mapped on the gene segment involved in the recombination (black letters in Fig. 3A and B) and by extending them in V and J region directions with the V and J gene segment sequences (green characters in Fig. 3A and B). *VD* and *DJ junction* sequences are also extracted from the reads in order to account for the role of the enzymatic processes in deleting or introducing nucleotides. These sequences are represented with red characters in Fig. 3A and B. If the mapping of the two mates is overlapped in the D region, no extension is needed between the end of the mapping of the first mate on the D gene segment and the start of the mapping of the other mate on the same gene segment. On the other side, in absence of overlap, the D gene segment is extended by concatenating, as for the V and J regions, the specific D gene segment sequence.

The initial *VDJ unmapped* reads are mapped taking advantage of Blast tool [40] on the new created references using default parameters. Every sequence is scored based on the number of reads supporting the nucleotide series. The most scored reference is considered as the main clone recombined sequence.

## Results

The proposed pipeline was applied to 5 MCL samples for which the quality was previously assessed. The mRNA was extracted using Allprep QUIAGEN Columns and then sequenced in 100 bp paired-end reads by means of Illumina HiSeq1000 technology. The number of reads (mate 1 and 2) obtained for each of the samples, in the following identified as Samples A, B, C, D and E, were respectively 40912861, 44147480, 44372085, 43524077 and 44139827. The results of the analyses performed using VDJSeq-Solver tool to characterise the V(D)J recombinations on the RNA-Seq data are reported in this section. The rearranged V(D)J sequences of the main clone for the considered samples were confirmed by wet lab tests via PCR. The research program that allowed to collect data was approved by the Institutional Review Board (IRB) of the University Hospital of Verona (Prog. 1933, Prot. 12516CE) and all clinical investigation have been conducted according to the Declaration of Helsinki. The use of anonymised samples (leftovers after clinical diagnostic procedures) was allowed by the IRB in absence of patients’ consent, whenever it was not reasonably possible to collect it since the patient could not be contacted, in compliance with the Italian law at the time of request.

**Fig 4:**
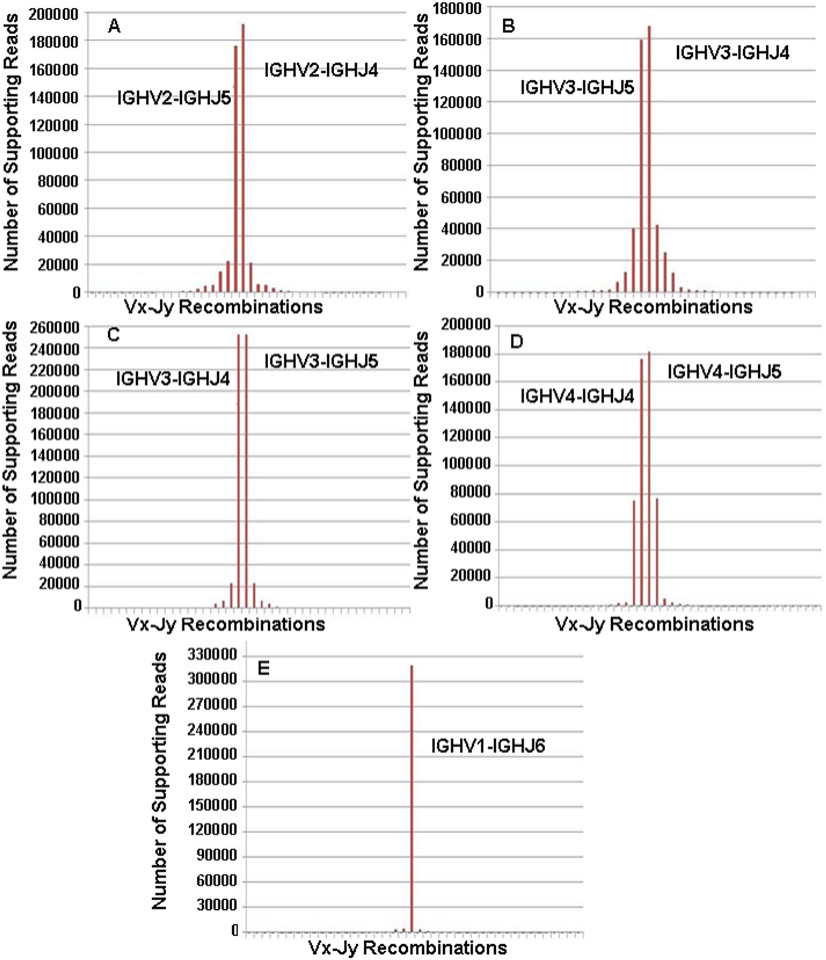
Supporting reads for the IGHV-IGHJ recombinations detected in the five MCL Samples. Subfigure A, B, C, D and E report respectively for Samples A, B, C, D, and E on the number of supporting reads for the detected IGHV-IGHJ recombinations. To better highlight the monoclonality feature of the distribution, the results are reported in terms of subgroups instead of gene segments or alleles. The most scored recombinations are identified in each sample with a label.

In Fig. 4A, B, C, D and E are reported respectively for Samples A, B, C, D and E, on the x-axis all the IGHV-IGHJ recombinations detected by VDJSeq-Solver tool in the different samples under examination with the relative number of supporting reads (y-axis). For clarity only the most scored recombinations have been explicitly identified in each sample with a label. Objective of this analysis is to highlight the monoclonal distribution characterising abnormal lymphocyte populations (as in the case of MCL) usually assessed in lab by means of the so called clonality tests. Note that, because of the polymorphic and homologous nature of BCR gene segments, alignment tools tend to map the same read in many chromosomal loci (multimapping) corresponding to different BCR gene segments. As such, to highlight the monoclonality of the distribution, in this case results are reported in terms of subgroups instead of gene segments or alleles. Indeed, sequence similarity appears generally to be less relevant across subgroups. In Fig. 4 Samples A, B, C and D are characterised by two recombinations being supported by a number of reads remarkably higher than those calculated for the others IGHV-IGHJ couples detected in the same samples. In particular, the two most scored rearrangements in each of these samples involve, for what is concerning IGHJ gene segments, IGHJ4 and IGHJ5 subgroups of which IGHJ4*02 and IGHJ5*02 gene segments are two members characterised by high similarity. On the other side, the two most supported recombinations in Samples A, B, C, D show the same IGHV subgroup: IGHV2 in Sample A, IGHV3 in Sample B, IGHV3 in Sample C and IGHV4 in Sample D. So, we can conclude that the two observed main clones are most likely the same one (according to biological insights). The number of supporting reads of the main clone in Samples A, B, C and D of Fig. 4 is consistently higher than that of the second most scored clone. Quantitatively the main clone is at least 2.4 times higher than the second one (as in Sample D) and reaches a factor of 11 in Sample C. For what is concerning Sample E, the main clone is only one, involving the IGHJ6 subgroup. Since for this subgroup there is no similarity with other subgroups, multimapping does not come into play in this case. Here, the main clone is supported by a number of reads which is 74 fold higher than that of the second clone. However, as will be reported later in this section, the identified main clones always correspond to the PCR validated ones for the considered samples.

**Fig 5:**
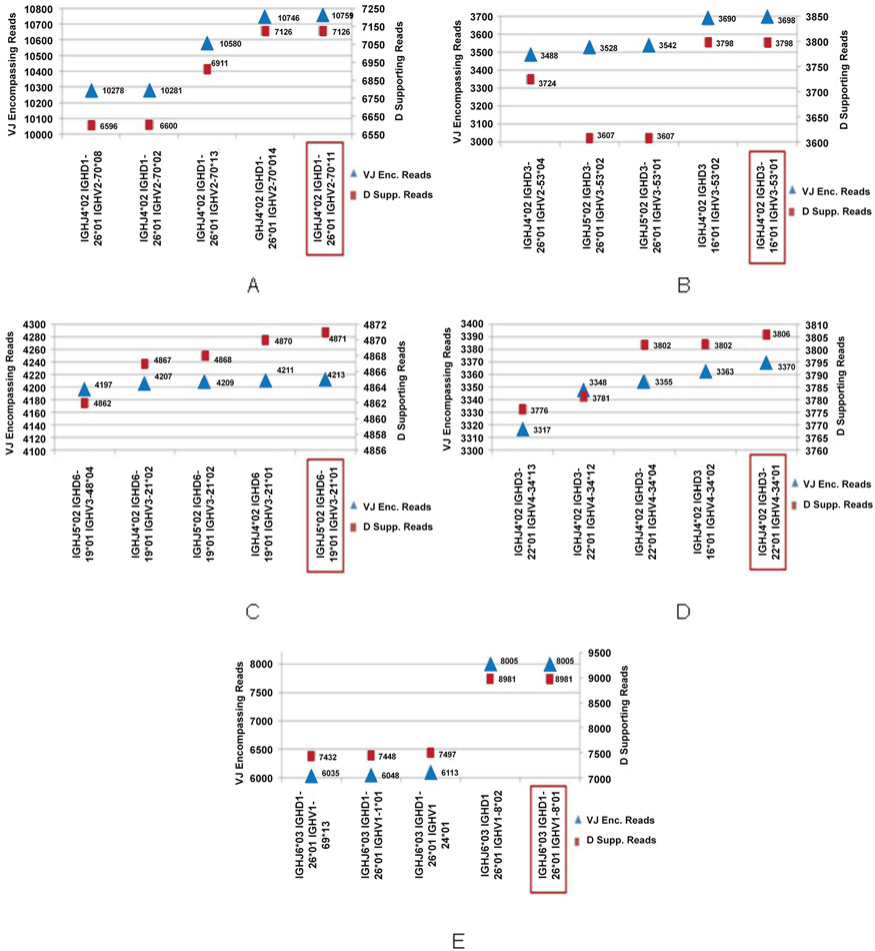
Supporting reads for the five clones most scored by reads in the MCL Samples. Subfigure A, B, C, D and E report respectively for Samples A, B, C, D, and E with blue triangles the number of reads supporting the different VJ recombinations on x-axis and with red boxes the number of mates supporting the D allele. For clarity two different scales have been selected to represent the obtained results: The one on the left of the graph is relative to VJ couples whereas the other to D allele. The main clone in each sample is highlighted with a rectangular box.

Fig. 5 shows the gene segments involved in the rearrangements for the recombinations detected in the samples under study. On the x-axis are shown the detected recombinations expressed as (J region—D region—V region). The notation depends on the type of region. For J regions, the encoding is: subgroup*allele. For D and V regions, the encoding is: *subgroup*-gene*allele. On the y-axis is instead reported the number of D or VJ supporting reads.

We present data concerning the five most supported clones by ranking in terms of the number of reads supporting them. In details, for each sample, we report for the detected recombinations the number of supporting reads for the VJ rearrangements as blue triangles, whereas the number of mates supporting the D alleles as red boxes. The main clone for each sample (highlighted in Fig. 5 with a rectangular box) has been identified as the one with the higher VJ score, since all the detected recombinations within a sample account for the same D gene segment. By looking at the detected recombinations of Fig. 5, we observe that the gene segments involved in all the recombinations belong to the same IGHV and IGHD subgroup in each of the analysed samples. For a given sample, the assignment of J spans instead two gene segments. For instance, in Sample B we have IGHJ5 in two out of the five most supported recombinations and IGHJ4 in three of them, including the most supported one. Overall, the most scored J gene segment always identifies the main clone. Starting from these observations and considering the impact of polymorphisms and homologies occurring in IGH gene segments alignment [41] (that cause the same mate to be mapped to different genes), we can assume that the identified recombinations are likely to belong to the same clone. The D gene segment detected for each specific recombination is the one characterised by the highest score after Shrimp alignment [42]. With respect to what observed for V and J gene segments, where monoclonality is highlighted when considering subgroups, D genes are maintained along all the recombinations characterising a given sample. Specifically: IGHD1-26*01 for Sample A, IGHD3-16*01 for Sample B, IGHD6-19*01 for Sample C, IGHD3-22*01 for Sample D and IGHD1-26*01 for Sample E (see Fig. 5). In the rearrangements characterising Sample A, shown in Fig. 5A, IGHV2 subgroup is the most reported alignment for the reads mapped on the V gene segments. In particular, all the rearrangements involve different polymorphic forms of IGHV2-70. On the other side, IGHJ4*02 is the most covered member among J gene segments. In Samples B and C (Fig. 5B and C), the alignments obtained for J gene segment involve both IGHJ5 and IGHJ4 subgroups (in particular IGHJ4*02 and IGHJ5*02). The homology pertaining to the last portion of IGHJ4*02 and IGHJ5*02 leads to small differences in the two nucleotide sequences. Thus, a mate aligning at the end of IGHJ4*02 gene segment will be probably aligned also to IGHJ5*02. Concerning V gene segment, in both B and C Samples, the IGHV3 subgroup is the only one involved in the identified recombinations. In particular in Sample B, IGHV3-53 is the only recombinant gene segment detected, whereas in Sample C the dominant gene segments are IGHV3-21 and IGHV3-48. In Samples D and E (see Fig. 5D and E) the J gene segments involved in the recombinations are respectively IGHJ4*02 and IGHJ6*03, whereas IGHV4 and IGHV1 are the subgroups identified for V gene segment alignments with essentially two members of the group: IGHV4-34 for Sample D and IGHV1-8 for Sample E. The predominance of specific subgroups for V, D and J gene segments and, in many cases, also of a specific member of these families in the rearrangements identified, strongly indicates that they account for the same main clone.

In order to validate the main clone sequences reconstructed by VDJSeq-Solver in the phase identified as *V(D)J Sequence Retrieving* we first obtained through PCR lab analysis the main clone sequences for the five MCL samples under examination. The nucleotide sequences of these main clones have been so provided as input of five freely available online tools that are able to detect V, D and J gene segments for a single sequence generally obtained with in lab experiments or thanks to single molecule sequencing technologies. Note that, as discussed in the *Introduction* Section, these tools are not capable to perform clonality analysis on RNA-Seq samples. Anyway, they can be used to validate our approach once the main clone has been obtained through PCR in laboratory. The tools used in this phase for the comparison are IMGT/V-QUEST integrated with IMGT/JunctionAnalysis program version 3.3.0 and reference directory release 201414-4 [23, 26–28, 30, 31], JOINSOLVER [29], VDJsolver 1.0 Server [25], SoDA [32] and iHMMune-align [24]. As shown in columns labelled as *PCR* in Table 1, there is not general consensus on the results of the alignment of the sequences extracted in laboratory. This is due to the heterogeneity of the algorithms used to perform the assignments. However, for all the samples under investigation, the tools mostly agreed on the gene segments assigned to the main clone sequences obtained through PCR (i.e. variations concern only allele polymorphisms). Exceptions are IMGT/V-QUEST [23, 26–28, 30, 31] in relation to the D gene segment assignment for Sample B as well as SoDA, and iHMMune-align, with respect to J gene segment assignment for Sample C, where they provided different gene segments callings with respect to PCR. With bold characters are highlighted the more divergent predictions provided for each of the analysed samples by the different tools.

**Table 1:** PCR and VDJSeq-Solver main clone sequences comparisons. Gene assignments for the PCR and the VDJSeq-Solver provided main clone sequences from five online tools.

		IGHV Gene Segment		IGHD Gene Segment		IGHJ Gene Segment	
Sample	Tool	PCR	RNA-Seq	PCR	RNA-Seq	PCR	RNA-Seq
A	IMGT/V-QUEST	IGHV2-70*11	IGHV2-70*11	IGHD1-26*01	IGHD1-26*01	IGHJ4*02	IGHJ4*02
A	JOINSOLVER	IGHV2-70*11	IGHV2-70*11	IGHD1-26*01	IGHD1-26*01	IGHJ4*02	IGHJ4*02
A	VDJsolver	IGHV2-70*11	IGHV2-70*11	IGHD1-26*01	IGHD1-26*01	IGHJ4*02	IGHJ4*02
A	SoDA	IGHV2-70*11	IGHV2-70*11	IGHD1-26*01	IGHD1-26*01	IGHJ4*02	IGHJ4*02
A	iHMMune-align	IGHV2-70*11	IGHV2-70*11	IGHD1-26*01	IGHD1-26*01	IGHJ4*02	IGHJ4*02
B	IMGT/V-QUEST	IGHV3-53*02	IGHV3-53*01	IGHD3-10*02	IGHD3-10*02	IGHJ4*02	IGHJ4*02
B	JOINSOLVER	IGHV3-53*02	IGHV3-47*03	IGHD3-16*02	IGHD3-16*02	IGHJ4*02	IGHJ4*02
B	VDJsolver	IGHV3-53*02	IGHV3-53*01	IGHD3-16*01	IGHD3-16*01	IGHJ4*02	IGHJ4*02
B	SoDA	IGHV3-53*01	IGHV3-53*01	IGHD3-16*01	IGHD3-16*01	IGHJ4*02	IGHJ4*02
B	iHMMune-align	IGHV3-53*01	IGHV3-53*01	IGHD3-16*02	**–**	IGHJ4*02	**–**
C	IMGT/V-QUEST	IGHV3-21*02	IGHV3-21*01	IHD6-19*01	IHD6-19*01	IGHJ5*02	IGHJ5*02
C	JOINSOLVER	IGHV3-11*02	IGHV3-11*02	IHD6-19*01	IHD6-19*01	IGHJ5*02	IGHJ5*02
C	VDJsolver	IGHV3-21*02	IGHV3-21*02	IHD6-19*01	IHD6-19*01	IGHJ5*02	IGHJ5*02
C	SoDA	IGHV3-21*02	IGHV3-21*01	IHD6-19*01	IHD6-19*01	IGHJ1*02	IGHJ1*02
C	iHMMune-align	IGHV3-21*01	IGHV3-21*01	IHD6-19*01	**–**	IGHJ4*02	**–**
D	IMGT/V-QUEST	IGHV4-34*01	IGHV4-34*01	IGHD3-22*01	IGHD3-22*01	IGHJ4*02	IGHJ4*02
D	JOINSOLVER	IGHV4-34*01	IGHV4-34*01	IGHD3-22*01	IGHD3-22*01	IGHJ4*02	IGHJ4*02
D	VDJsolver	IGHV4-34*01	IGHV4-34*01	IGHD3-22*01	IGHD3-22*01	IGHJ4*02	IGHJ4*02
D	SoDA	IGHV4-34*01	IGHV4-34*01	IGHD3-22*01	IGHD3-22*01	IGHJ4*02	IGHJ4*02
D	iHMMune-align	IGHV4-34*01	IGHV4-34*01	IGHD3-22*01	IGHD3-22*01	IGHJ4*02	IGHJ4*02
E	IMGT/V-QUEST	IGHV1-8*01	IGHV1-8*01	IGHD1-26*01	IGHD1-26*01	IGHJ6*03	IGHJ6*03
E	JOINSOLVER	IGHV1-8*01	IGHV1-8*01	IGHD1-26*01	IGHD1-26*01	IGHJ6*03	IGHJ6*03
E	VDJsolver	IGHV1-8*01	IGHV1-8*01	IGHD1-26*01	IGHD1-26*01	IGHJ6*03	IGHJ6*03
E	SoDA	IGHV1-8*01	IGHV1-8*01	IGHD1-26*01	IGHD1-26*01	IGHJ6*03	IGHJ6*03
E	iHMMune-align	IGHV1-8*01	IGHV1-8*01	IGHD1-26*01	IGHD1-26*01	IGHJ6*03	IGHJ6*03

**Fig 6:**
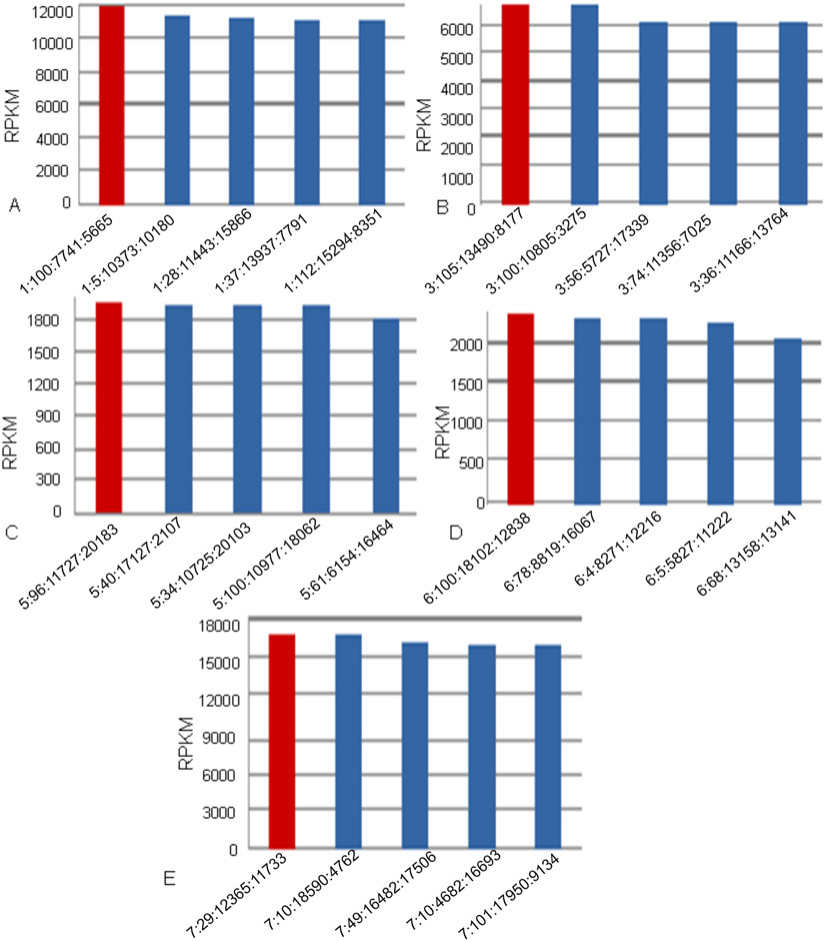
RPKM values for the five main clone sequences with higher RPKM values in the analysed MCL Samples. Subfigure A, B, C, D and E report respectively for Samples A, B, C, D, and E on the y-axis the RPKM values for the five most scored virtual references on x-axis. Each of the reference associated to the detected main clone is labelled with the unique numeric identifier of the read from which the sequence has been reconstructed. In red are highligted the top scored sequences whereas in blue the following ones.

As VDJSeq-Solver is able to provide the V(D)J sequence of the main clone by means of the *V(D)J Sequence Retrieving* algorithm, we validated the obtained sequences with the ones provided by PCR. The proposed tool scores the virtual references that represent the reconstructed sequences using a RPKM measure. Fig. 6 reports, respectively for Samples A, B, C, D and E, on the y-axis the RPKM values for the five most scored virtual references on x-axis. Each reference is labelled in Fig. 6 by means of the unique numeric identifier of the read from which it has been retrieved. The RPKM values were obtained in this phase by mapping the initially *VDJ unmapped* reads on the reconstructed virtual references using Blast [40]. The most scored sequence for each of the analysed sample is characterised by the highest percentage of similarity with respect to the sequence provided by PCR analysis and it is represented in Fig. 6 with a red bar.

**Fig 7:**
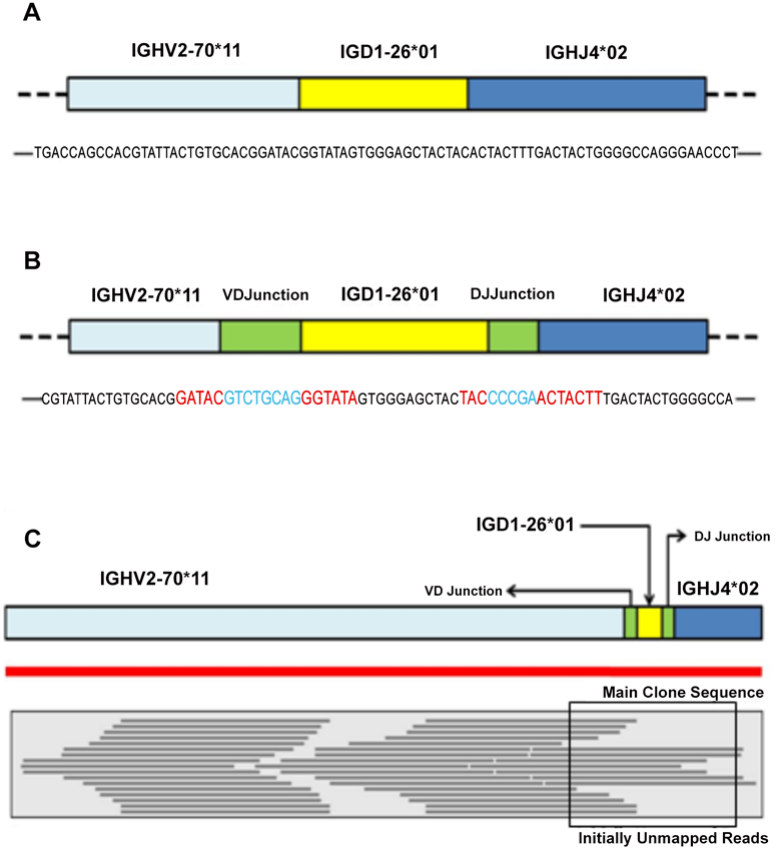
Sample A main clone sequence analysis. In Subfigure A and B are respectively shown the main clone V(D)J sequence in absence and in presence of enzymatic processes. The blue nucleotides are those introduced by different enzymes, such as the TdT, in the VD and DJ junctions during the rearrangement, whereas the red ones those deleted. Subfigure C shows instead how the initially VDJ unmapped reads are aligned on the main clone sequence provided by VDJSeq-Solver tool.

The role of the enzymatic processes during V(D)J recombination can be highlighted by comparing the main clone sequences obtained both in lab with PCR and in silico thanks to VDJSeq-Solver tool with those deriving by the simple V, D and J gene segments concatenation. In Fig. 7 we graphically show this effect as well as the mapping of the initially *VDJ unmapped* reads on the reconstructed V(D)J main clone sequence for Sample A. As depicted in Fig. 7A, in absence of the enzymatic processes the three V, D and J gene segments are joined together without insertions or deletions of nucleotides. Thus, the main clone sequence is the perfect concatenation of V, D and J nucleotide sequences. Fig. 7B shows how enzymatic processes act on the main clone sequence detected by VDJSeq-Solver in Sample A. In particular, the light blue nucleotides are those introduced in the so called *VD Junction* and *DJ Junction* (corresponding to the green section on the upper side of Fig. 7B whereas the red ones represent those that have been deleted. In Fig. 7C is shown the arrangement of the initially *VDJ unmapped* reads on the main clone sequence extracted using VDJSeq-Solver tool. This disposition fits the ladder-like pattern that has been proven to characterise biologically validated transcript rearrangements [43].

**Fig 8:**
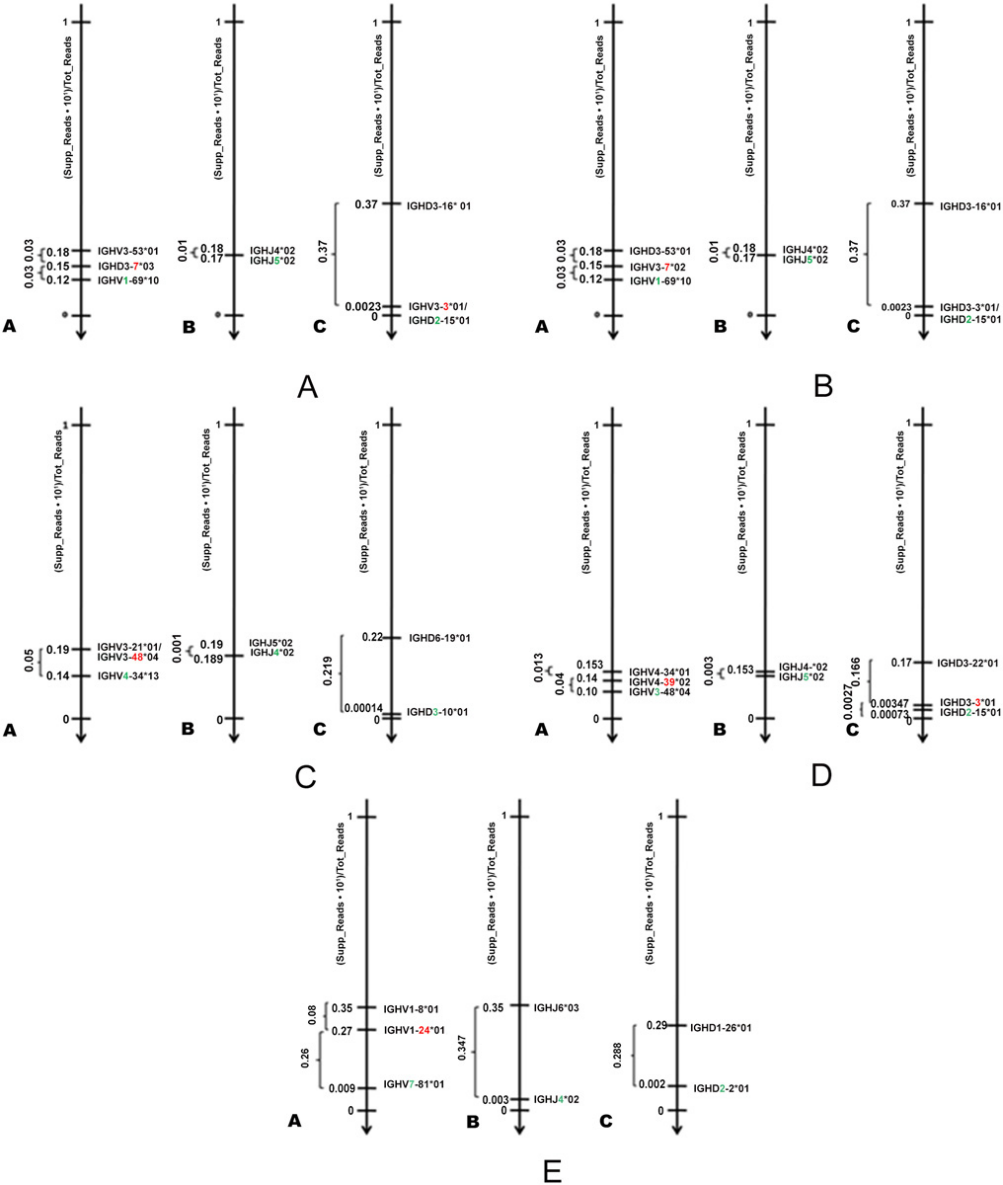
RPKM-like distance between the main clone and the other most supported clones having different gene segments and subgroups in the five MCL Samples. Subfigure A, B, C, D and E report respectively for Samples A, B, C, D, and E on the differences in terms of normalized read counts between the main clone detected in the sample and nearest clones characterised by different gene segments and subgroups.

An accurate read mapping, able to account for the impact of enzymatic processes, is functional to one of the main objective of the proposed tool, that is distinguishing between clones characterised by different subgroups or gene segments. To this purpose we widely investigate the difference, in terms of normalized reads, between the main clone and other mostly supported clones characterised by different gene segments and different subgroups proving that effectively the distance between two clones reaches its maximum value when the subgroup changes. Fig. 8 highlights the difference, in terms of normalized reads, between the main clone and other most supported clones characterised by different gene segments and different subgroups. For instance, in Fig. 8A, the main clone is IGHV2-70*11 (subgroup V2, gene 70 and allele 11), which has a normalized read count of 0.53. The second most supported clone, having a different gene, is IGHV2-5*08 (subgroup V2, gene 5 and allele 8) which has a normalized read count of 0.42. Finally, the most supported clone belonging to a different subgroup is IGHV3-48*04 (subgroup V3, gene 48 and allele 4). The normalized read count we used is a RPKM-like representation, where, compared to RPKM, we do not normalize for the size of transcribed regions V, D, J. The reason why we used a RPKM-like quantification is because we wanted to quantify how much a sequence is supported independently from the sample coverage. However, RPKM as it is cannot be used in this case. Indeed, being adopted to estimate the expression levels, RPKM normalises by the length of the transcript to compensate for the fact that reads are distributed almost uniformly along the transcript, whose size is in general a multiple of the mate length. However, in the particular case of V(D)J transcripts, the length of the transcripts is comparable and in some cases smaller (like regions D and J) than a single mate. Hence, normalising with respect to transcript length (which is lower than a single mate) would lead to incorrect expression level comparisons between the different clones.

**Fig 9:**
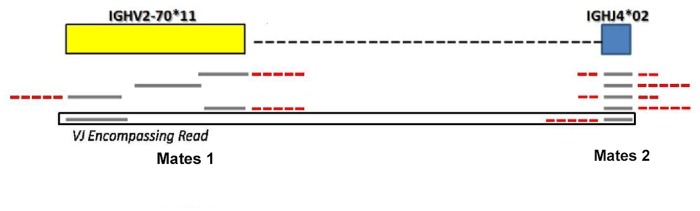
VJ Encompassing Read definition. In Figure an example of encompassing VJ read is reported. An encompassing VJ read is detected if the two mates of a read are mapped totally or not respectively on a V and on a J gene segment. The number of encompassing reads supporting each recombination allows to sort the different rearrangements detected.

Furthermore, as depicted in Fig. 9, the *VJ encompassing* reads are only the ones for which a mate mapping in J and the other in V region is found. Thus, the total number of reads does not represent the total number of reads mapping in the V region, causing the normalization done by the standard RPKM formula (that is by the size of V region) being unsuitable (i.e. leading to an underestimation of the RPKM expression). In the light of these considerations the RPKM is calculated as follows:

$$\begin{array}{c} RPKM_{VDJ}=\frac{VJenc\text{\_}reads}{Tot\text{\_}reads}*10^{9} \end{array}$$ (1)

Having removed this normalization, the RPKM-like measure we adopted provides a viable way to sort the obtained recombinations.

**Fig 10:**
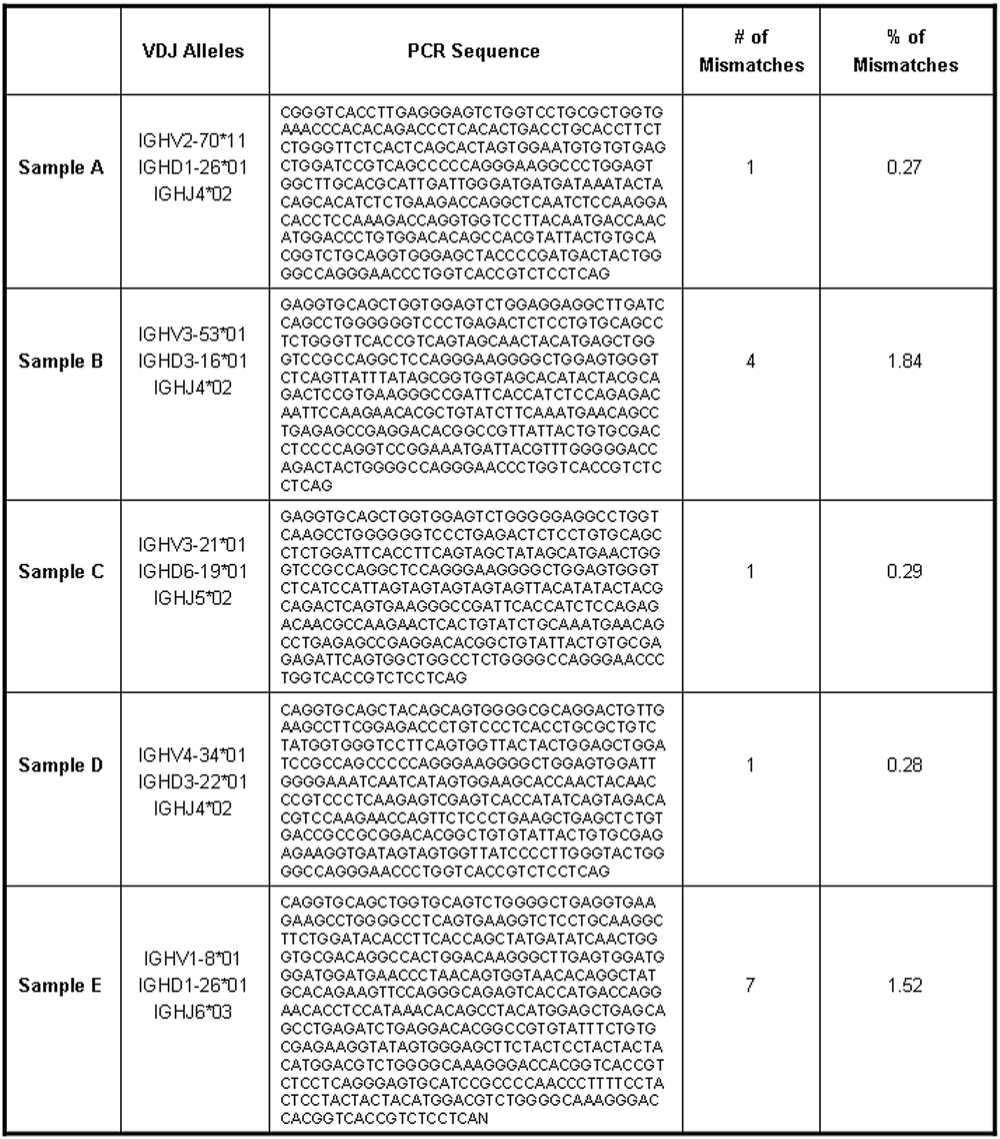
Data relative to the main clones detected by VDJSeq-Solver tool in the five MCL Samples. In each line are reported, respectively for Samples A, B, C, D and E, the V, D and J gene segments involved in the main clone recombination, the relative V(D)J rearranged sequence, the number of mismatches identified in this sequence with respect to the PCR validated sequence and the percentage ratio between the number of mismatches and the sequence length.

The most supported sequence for each sample has been afterwards compared to the one extracted via PCR in laboratory showing a number of mismatches always lower than 7 and a percentage error lower than 1,85. Fig. 10 depicts, for each of the samples under examination, data relative to our main clone. Starting from the second column, in Fig. 10 are reported respectively: The gene segments involved in the recombination, the extracted sequence, the number of mismatches found in our sequence with respect to the sequence obtained in laboratory and the ratio between the number of mismatches detected in our sequence and the sequence length. In order to establish whether the mismatches detected in the main clone sequences could impact the results given by the previous cited five online tools, we inserted our sequences in the same tools. Indeed, even if the number of mismatches of the in-silico obtained sequences are not high, our interest is to test if their positions are capable to account for different gene segments predictions. In Table 1, columns labelled as *RNA-Seq*, are reported the results. As noticed before with the PCR validated sequences of Samples B and C (see Table 1 columns labelled as *PCR*) also for our sequences belonging to the same samples IMGT/V-QUEST and JOINSOLVER (respectively for D and V gene segments assignments in Sample B), SoDA and JOINSOLVER (respectively for J and V gene segments assignments in Sample C) provided discordant predictions. Furthermore iHMMune-align was not able to identify an assignment for D and J gene segments in Samples B and C even when the required number of D matching nucleotides was set to the minimum allowed threshold. In bold letters are highlighted the more divergent and the null predictions provided for each of the analysed samples by the different tools. However, looking at Table 1, it is worth noting that the assignments for our sequences and the PCR validated sequences, if present, are in the most of the cases identical within the sample at the gene segments level, proving that the mismatches detected in our sequences, with respect to the PCR provided ones, are not capable to account for different assignments by the considered tools.

**Fig 11:**
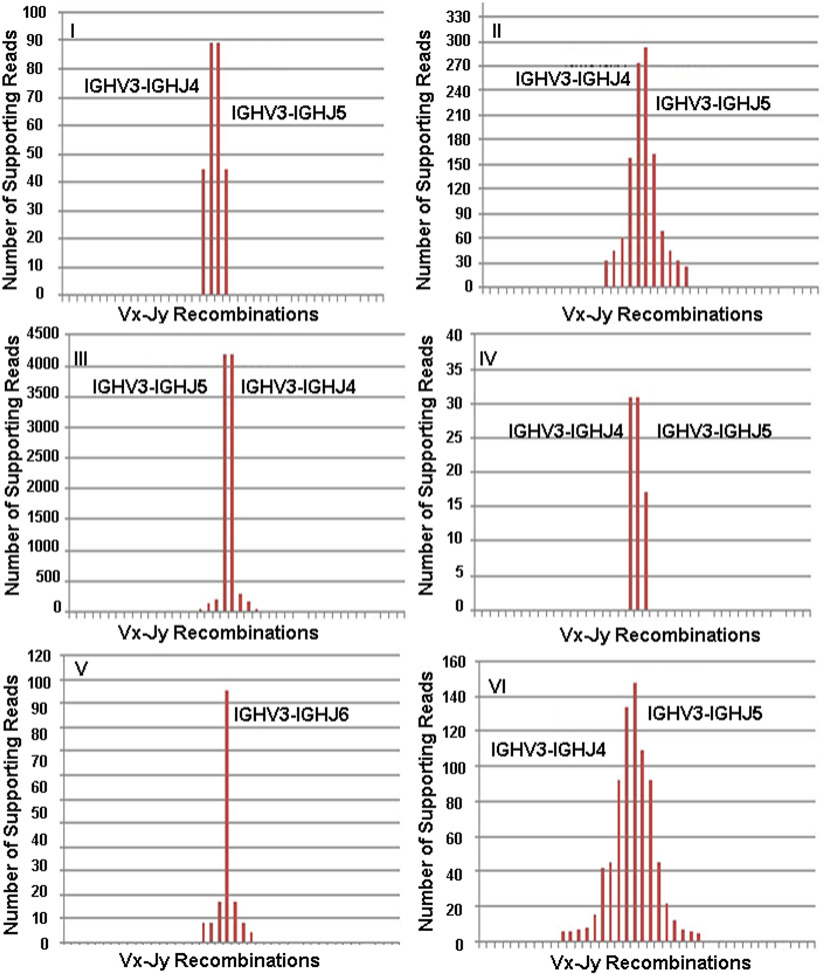
Supporting reads for the detected IGHV-IGHJ recombinations in six DLBCL Samples from TCGA. Subfigure A, B, C, D and E report respectively for Samples I, II, III, IV, V and VI on the number of supporting reads for the detected IGHV-IGHJ recombinations.

**Fig 12:**
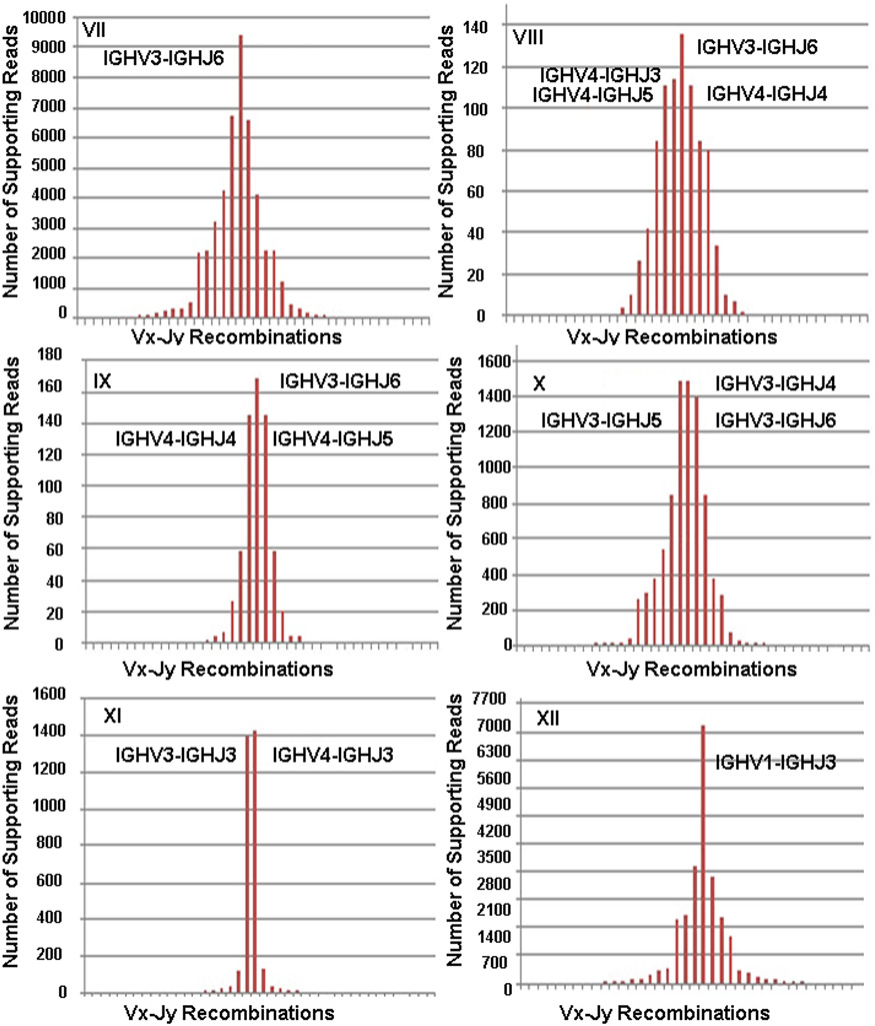
Supporting reads for the detected IGHV-IGHJ recombinations in six DLBCL Samples from TCGA. Subfigure A, B, C, D and E report respectively for Samples VII, VIII, IX, X, XI and XII on the number of supporting reads for the detected IGHV-IGHJ recombinations.

In order to further test VDJSeq-Solver performances on public datasets we applied the proposed pipeline on twelve paired-end 50bp long RNA-Seq DLBCL samples downloaded from TCGA. As widely discussed in [44] the predominance of BCR harbouring a specific clonal rearrangement of IG gene segments was expected as output of our analysis. Figs. 11 and 12 report on the IGHV-IGHJ recombinations detected in the analysed samples by VDJSeq-Solver tool, with the relative number of supporting reads on the y-axis. In particular 7 out of 12 samples (i.e Samples I, II, III, IV, V, VI of Fig. 11 and Sample VII of Fig. 12) are characterised by a predominant rearrangement involving IGHV3, IGHV4, IGHJ4 and IGHJ6 subgroups according to [44]. In Samples I, II, III, IV and VI the two main recombinations account for the same clone since involving IGHJ4 and IGHJ5 subgroups. It is worth noting that in these samples the first detected clone is well separated, in terms of reads coverage, from the other identified clones. Moreover, 3 out of 12 samples (i.e. Samples VIII, XIX and X of Fig. 11) show a main rearrangement in agreement with [44] even though a more polyclonal background is evident. Finally 2 out of 12 samples reveal a main clone characterised by different subgroups with respect to those defined as predominant in [44]. As confirmed there, also the other subgroups can partecipate, although to a lesser extent, in the main clone V(D)J rearrangement.

## Discussion

Identifying the presence of a clonal population of lymphocytes and providing the precise sequence information regarding BCR gene segments usage are two important goals of modern diagnostic molecular hematopathology. Usually these two tasks are performed separately, and both have limitations in their current output. Moreover, the identification of all B-cell clones in a given sample might be an important tool in immunological research, and this task is rather laborious using the current technology. BCR and TCR gene segments can be amplified by means of PCR using consensus oligonucleotide primers matching to conserved regions [45–47] or multiple primers [48, 49]. In a polyclonal lymphocyte population, this kind of amplification produces multiple products, which tend to distribute in a gaussian shape when analysed by electrophoresis. This rather reproducible distribution is exploited to detect abnormal expansion of single clonal populations, which result in one (or more) preferentially amplified products, visible as sharp peaks over the gaussian curve. Due to the ease of set-up and low turnaround time, PCR-based clonality tests are very popular in diagnostic hematopathology, and have largely replaced more laborious techniques such as Southern blotting [50, 51]. However, these techniques suffer from a few major drawbacks. First, due to the difficulty in designing a proper primer set, that is able to amplify all possible gene segments rearrangements (including variations caused by somatic hypermutations), a compromise has to be reached between sensitivity and specificity of tests. Second, due to amplification biases and to the intrinsically non-quantitative nature of PCR techniques, the interpretation of the results is based on visual inspection by an expert operator [49, 52–54], while the application of objective interpretation algorithms is strongly discouraged [54]. As a consequence, current protocols suffer from amplification biases and are inherently non-quantitative, leaving ample margin to subjective interpretation of results, especially concerning the determination of clonality. Moreover, they only describe what is chosen as the main clonal population, providing an incomplete picture of the immunological background of leukemias and lymphomas. The BCR and TCR of neoplastic lymphocytes however are not just clonal markers. Their expression is usually retained in cells that have undergone a strong selective pressure and are therefore supposed to have a *lean* phenotype. This fact might suggest that these protein complexes bear an advantage to neoplastic cells, and evidence for this is accumulating, at least for B-cell malignancies. The most widely studied malignancy in this regard is B-CLL, which is also the commonest leukemia in the western world [55]. The pathogenetic role of the BCR in B-CLL is supported by a wealth of evidence: a) The use of IGHV, IGHD and IGHJ gene segments is strongly biased compared to the expected distribution [9–16]; b) although less studied, also the use of IGLV and IGLJ gene segments is also skewed [17, 18]; c) many B-CLL cases bear identical or quasi-identical Heavy Third Complementary Determining regions (VH CDR3) that are BCR regions strongly determinant for antigen specificity [56–60]; d) many BCR pathway members are active in B-CLL cells [61]; e) clinical course is correlated to the rate of somatic hypermutation of BCR gene segments [19, 20]; f) the presence or absence of autoimmune phenomena correlates with the level of somatic hypermutations [62, 63]. Recently, some of these phenomena have been investigated also in other B-cell malignancies, including MCL [64–67] and diffuse large B-cell lymphoma [68, 69]. Taken together, these data demonstrate that knowing the precise sequence of rearranged BCR gene segments provides lots of useful information both from an investigative and from a clinical point of view. Thanks to its quantitative nature, RNA-Seq seems to be the ideal approach to identify, quantify and provide sequence information regarding candidate clones in a complex population of lymphocytes. The high transcription rate of BCR gene segments makes it easy to obtain very high sequence coverage and get rid of most of the background caused by non-rearranged gene segments. Easy as it might seem, this approach needs an effective computational method to be carried out. In fact, the complex rearrangement of BCR gene segments results in unique RNAs which cannot simply be mapped to the reference genome. To our knowledge, an approach dealing with the task of identifying these RNAs is completely lacking. A few recent papers describe applications of NGS technologies to the detection of IGHV repertoire [33, 69, 70], and one focuses on possible diagnostic uses for these technologies [71]. However, all these publications deal with the application of NGS technology to deep sequencing of amplified PCR products, while none of them use native RNA-Seq data.

## Conclusions

In this paper we present a novel algorithm and tool, namely VDJSeq-Solver, suitable for short/medium-read paired-end approaches, to identify the main clonal population in complex cell mixtures, and to provide precise sequence information regarding BCR gene segments. Homologies and polymorphisms typical of IGH gene segments [41] represent major issues that must be solved to implement an effective identification flow. These features cause indeed alignment tools to report different mapping for the same read if the number of mismatches allowed during the mapping is not conservative enough. The choice of the proper thresholds is not trivial as the lower are their values, the higher will be the alignment execution time. During all the alignments performed with Blast [40] we used default parameters as these allow to distinguish sequences characterised by a percentage of similarity equal to 99. We adopted some adjustments to account for this accuracy limitation. Specifically, in order to avoid the impact of multimapping due to homologies inside the same gene segment, which may introduce an overestimation of the reads supporting a specific recombination, reads accounting for the same recombination at different positions are considered only a time in the calculation of the reads supporting the rearrangement in the *VJ couples sorted occurrence calculation* phase. On the other side, if a read supports different recombinations, due to polymorphisms and homologies, that read is not removed because of the uncertainty related to its corrected assignment. Therefore, a sorting on the basis of the number of *VJ encompassing* reads supporting the detected recombinations is performed. As a consequence, the most supported couple is identified as that characterising the main clone. Similar issues must be dealt in the *D alleles individuation* step. Here, the reduced dimension of the set of input reads is computationally affordable for Shrimp aligner [42] usage. Shrimp works better in presence of a large amount of polymorphisms in the reference genome, by specifying a seed region to be searched in the D alleles.

VDJSeq-Solver was able to identify the main clones in the test-sets composed of five MCL and twelve DLBCL samples. Future work will be devoted to adopt a larger validation set including non-neoplastic samples. The use of several non-neoplastic samples should provide indeed the necessary background information to develop a diagnostic test. We envision a number of exploitation paths for the proposed methodology, such as: i) Identification and characterisation of sub-clones or divergent clones in a neoplastic population and follow them up over time; ii) identification of clonality of light chains, which should provide helpful information both for diagnostic purposes and for immunology research; iii) identification of T-cell receptor rearrangements, with obvious impact on the diagnostic approaches of T-cell lymphomas; iv) coupling to gene-signatures defining specific World Health Organization (WHO) entities, bringing molecular diagnostic hematopathology to a previously unthinkable degree of precision.
